# Modulation of Inhibitory Corticospinal Circuits Induced by a Nocebo Procedure in Motor Performance

**DOI:** 10.1371/journal.pone.0125223

**Published:** 2015-04-29

**Authors:** Mehran Emadi Andani, Michele Tinazzi, Nicole Corsi, Mirta Fiorio

**Affiliations:** 1 Department of Neurological and Movement Sciences, University of Verona, Verona, Italy; 2 Department of Biomedical Engineering, University of Isfahan, Isfahan, Iran; University of Ottawa, CANADA

## Abstract

As recently demonstrated, a placebo procedure in motor performance increases force production and changes the excitability of the corticospinal system, by enhancing the amplitude of the motor evoked potentials (MEP) and reducing the duration of the cortical silent period (CSP). However, it is not clear whether these neurophysiological changes are related to the behavioural outcome (increased force) or to a general effect of expectation. To clarify this, we investigated the nocebo effect, in which the induced expectation decreases force production. Two groups of healthy volunteers (experimental and control) performed a motor task by pressing a piston with the right index finger. To induce a nocebo effect in the experimental group, low frequency transcutaneous electrical nerve stimulation (TENS) was applied over the index finger with instructions of its detrimental effects on force. To condition the subjects, the visual feedback on their force level was surreptitiously reduced after TENS. Results showed that the experimental group reduced the force, felt weaker and expected a worse performance than the control group, who was not suggested about TENS. By applying transcranial magnetic stimulation over the primary motor cortex, we found that while MEP amplitude remained stable throughout the procedure in both groups, the CSP duration was shorter in the experimental group after the nocebo procedure. The CSP reduction resembled previous findings on the placebo effect, suggesting that expectation of change in performance diminishes the inhibitory activation of the primary motor cortex, independently of the behavioural outcome.

## Introduction

The nocebo effect is a detrimental outcome that follows the application of an inert treatment. It is commonly considered as the negative counterpart of the positive placebo effect, in which a benefit is obtained. With regard to pain, nocebo and placebo effects not only result in opposite outcomes (hyperalgesia vs. analgesia, respectively), but also subtend slightly opposite neural mechanisms [1, 2]. For instance, nocebo hyperalgesia is associated with increased activation in the spinal cord [3] and in brain regions involved in pain processing, like the thalamus, the insula, the prefrontal cortex, the anterior cingulate cortex [4, 5]. Moreover, nocebo hyperalgesia seems to be specifically associated to the cholecystokinin system, which is involved in the modulation of anxiety [6, 7]. With regards to placebo analgesia, a reduced activation was observed in the spinal cord [8] and in pain-processing brain regions [9]. Furthermore, placebo analgesia is related to opioid and non-opioid systems [6, 7, 10].

Research has recently started to elucidate the behavioral and neurophysiological correlates of these effects in the motor domain, giving greatest attention to placebos [11–13]. In this regard, beside a placebo-induced enhancement of force, we recently documented corticospinal changes following a placebo procedure, as shown by increased amplitude of the motor evoked potentials (MEP) and reduced duration of the cortical silent period (CSP) [14].

Up to now, only few studies demonstrated a worsening of motor performance following a nocebo procedure. Team-sport athletes showed a reduction of speed during performance following the administration of an inert substance together with negative information about its effects [15]. Even in nonathletes increase of muscle work during a leg extension exercise was prevented by inducing a negative belief about the effect of sham electrical stimulation over the leg muscles [16]. The aim of the present study is to uncover the behavioral and neurophysiological correlates of the nocebo effect in motor performance. To this end, we took advantage from a previously developed paradigm [14], adequately adapted to induce nocebo effects on force production, and applied transcranial magnetic stimulation to measure MEP and CSP. While at the behavioral level it is reasonable to expect a nocebo-induced reduction of force, the prediction is less obvious for the neurophysiological correlates. If nocebo effects in the motor system are associated to the behavioral outcome, we should expect opposite results to those previously described for placebo [14], i.e., a decrease of corticospinal excitability. However, differently from MEP amplitude, CSP duration is not necessarily related to changes in the amount of force [17–19] and therefore it could be modulated even independently from the behavioral outcome.

## Materials and Methods

### Participants

Thirty-two healthy volunteers participated in the study and were randomly divided into two groups: 17 subjects (all but two right-handed) entered the experimental group (7 women and 10 men; mean age, 23.3 ± 2.9 years old) and 15 subjects (all but two right-handed) entered the control group (9 women and 6 men; mean age, 22.1 ± 2.2 years old). The two groups did not statistically differ for age (independent samples t-test, t_(30)_ = 1.347; p = 0.188). Moreover, the two groups were comparable for gender distribution (Chi-square test, χ^2^ = 1.129, df = 1, p = 0.288).

Participants were recruited from the student population of the University of Verona. Before starting the study, all the subjects received an information sheet in which the experimental procedure and the TMS technique were explained in detail—the real purposes of the study, instead, were explained only at the end of the whole experimental procedure. After having read the information sheet, subjects signed a written informed consent form in which they also declared to have no history of neurological, psychiatric, or other medical problems. The study was conducted according to the principles expressed in the Declaration of Helsinki and was approved by the ethical committee of the Department of Neurological and Movement Sciences, University of Verona, Italy (approval number 47921).

### Motor task

**Fig 1:**
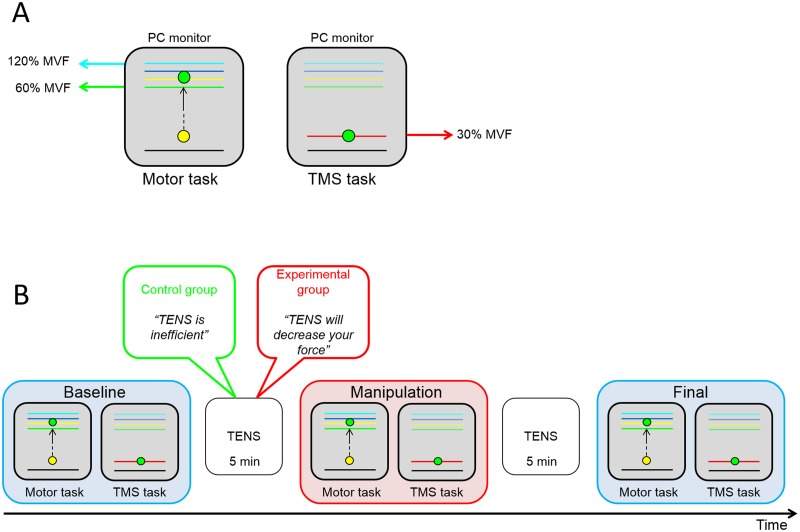
Schematic representation of the experimental set-up and protocol. A) In the left panel, a representation of the motor task is shown. On the PC monitor there was a starting line (black line), a target zone (colored lines) and a cursor (yellow and green dot). The lowest line of the target zone (green) represented the 60% of the subject’s MVF, while the highest line (light blue) represented the 120% of MVF, as measured for each participant in the calibration phase at the very beginning of the experiment. The task was to press a piston with the right index finger as strong as possible in order to reach the higher lines of the target zone. The pressures against the piston were recorded by a force transducer and converted in vertical displacements of the cursor. When the cursor entered the target zone, it changed from yellow to green. In the right panel, a representation of the TMS task is shown. The red line represents the 30% of MVF as measured in the calibration phase. Subjects had to maintain the cursor on the red line until the end of the trial. B) Timeline of the experimental protocol with a representation of the sessions sequence (baseline, manipulation and final) and TENS application. As schematically shown in the picture, the two groups of participants (control and experimental) received different verbal information about the effects of TENS. Moreover, in the manipulation session the two groups underwent different procedures: the experimental group performed the motor task with a surreptitious reduction of the cursor’s excursion range (conditioning procedure), while the control group performed the motor task without any reduction.

The same motor task was used as described in a previous placebo study [14], consisting in abduction movements of the right index finger against a piston connected to a force transducer (DS BC302). The contribution of the first dorsal interosseous muscle (FDI) was isolated by restricting the hand with a holder. In real-time, finger pressures against the piston were linearly converted into vertical displacements of a cursor shown on a PC monitor. In this way, the cursor acted as visual feedback on the amount of force exerted by the subject. For each subject the maximal voluntary force (MVF) was measured before starting the experimental sessions. This value was then used to scale the vertical displacements of the cursor on the PC monitor during the task. Subjects could initiate each single trial by pressing the mouse with the left hand when a “START” signal appeared on the screen and were then asked to press the piston as strongly as possible in order to move the cursor upwards within a target zone made of four colored horizontal lines. Unbeknown to the subject, the lines represented the 60%, 80%, 100% and 120% of the subject’s MVF (Fig 1A). In this way, there was enough margin to reduce the cursor’s excursion range below the subject’s MVF (100%), thus producing a negative feedback of worse performance in the manipulation session, as to condition the subjects to reduce their force levels (see below). The task consisted of 50 trials of 1100 ms each. An initial short training of 5 trials allowed the subjects to familiarize with the task.

### Procedure

The protocol included three sessions: baseline, manipulation and final (Fig 1B). The baseline and final sessions were identical in both groups and consisted in the execution of the motor task described above. These two sessions allowed comparing subjects’ performance before and after the nocebo manipulation.

Nocebo effects were obtained by applying an inert treatment (10 Hz transcutaneous electrical nerve stimulation, TENS) for 5 minutes over the region of the FDI belly. The intensity of TENS was adjusted until the subject reported a slight sensation without muscle contraction. Subjects were also asked to report whether TENS was painful or uncomfortable. None of the participants reported these sensations. Participants of the experimental group were told that TENS could reduce the recruitment of muscle fibers, thereby decreasing force production. Because of the cutaneous sensation perceived by the subjects over the region of the hand muscle involved in the task, TENS can be expected to manipulate the subject’s belief of bad motor performance. In order to reinforce the subjects’ belief about the effects of TENS, the experimental group underwent a conditioning phase. A pre-determined, surreptitious reduction of the cursor’s excursion range was introduced stepwise. More precisely, after TENS, the motor task was executed again, but this time unbeknown to the subjects an attenuation coefficient was introduced and the excursion of the cursor was gradually decreased in steps of 0.0029 from trial 1 to trial 35 and remained stable until the end of the session (from trial 36 to trial 50). Consequently, by applying the same amount of force as in the baseline, the participants of the experimental group could see the cursor achieving lower lines of the target zone than before, and therefore believed to be weaker because of TENS.

Before starting the final session, TENS was applied again together with verbal suggestion of worse motor performance. Subjects then repeated the motor task (50 trials), but this time without any manipulation, that is without the reduction of the cursor’s excursion range.

The same motor task was performed by the subjects of the control group, who also underwent the TENS application as described above, but with different verbal information. In particular, these subjects were clearly told that they have been assigned to a control group in which TENS was completely inert in affecting force. They executed the motor task three times, like the experimental group, but without reduction of the cursor’s displacements in the manipulation session.

For each subject, the whole experiment took about 1.5 hours to be completed. Participants were tested at different times during the day, starting from 9.00am to 5.00pm. In the experimental group, 9 subjects were tested in the morning (9.00am-12.00am) and 8 subjects were tested in the afternoon (1.00pm-5.00pm). In the control group, 9 subjects were tested in the morning (9.00am-12.00am) and 6 subjects were tested in the afternoon (1.00pm-5.00pm). By analyzing the distribution of the subjects tested in the morning and in the afternoon, we found no differences between the two groups (Chi-square test, χ^2^ = 0.161, df = 1, p = 0.688).

### Behavioral data

Force was assessed in two ways. The mean value of the peak force amplitude in the 50 trials of each session (Force_peak_) was normalized to the maximum voluntary force (MVF). This index was defined as follows:

$$Normalized\;Force_{peak}\;=\;\frac{Force_{peak}}{MVF}\;\times 100\%$$

Additionally, the percentage of strong pressures (Strong_press_) in each session was calculated with the formula:

$$Strong_{press}\;=\;\frac{N_{strong\;trials}}{N_{tot\;trials}}\;\times 100\%$$

where *N*_*tot trials*_ is the total number of trials in each session (i.e., 50) and *N*_*strong trials*_ is the number of trials in which the peak force was above the mean value computed in the baseline.

In order to monitor fatigue throughout the procedure, we extracted a fatigue index (FI) from our data [adapted from 20, 21]:

$$FI=\left(1-\frac{AUF}{MVF\times t}\right)\;\times 100\%$$

where *AUF* is the area under force (i.e., integral of force against time) in each trial, *MVF* is the maximal voluntary force and *t* is the duration of a trial. FI is entailed between 0 and 100. Differently from previous studies in which fatigue was measured in a single, continuous task lasting at least 30 sec [20, 21], our task was a combination of 50 discrete trials lasting 1.1 sec. Hence, in order to quantify fatigue during each experimental sessions, we computed the mean value of FI of all the trials, (i.e., by averaging FI from trial 1 to 50). Small values of FI indicate no fatigue, conversely big values of FI indicate fatigue.

### Subjective data

Subjective evaluation took into account different aspects. *i) Expectation level*. After TENS application, subjects were asked to judge whether they expected a change performance compared to baseline. To this purpose, a 7-points numeric rating scale (NRS), ranging from -3 (expectation of much worse performance) to +3 (expectation of much better performance) was administered. *ii) Judgment of TENS efficacy*. After the execution of the motor task in the manipulation and final sessions, subjects were asked to report whether TENS was effective in reducing force, by means of a 10 cm visual analog scale (VAS), ranging from 0 (not effective) to 10 (very effective). *iii) Perception of force*. Soon after completion of the motor task in the baseline session, subjects were asked to estimate the level of perceived force during the execution of the task by means of a 10 cm VAS scale, ranging from 0 (very weak) to 10 (very strong). Afterward, in the other two sessions (manipulation and final), subjects were asked to judge how strong they felt with respect to the baseline session by means of 7-points NRS, ranging from -3 (much weaker) to +3 (much stronger). *iv) Sense of extent*. Subjective sense of effort was measured in each session after the execution of the motor task, by means of the Borg scale [22], ranging from 6 (rest) to 20 (maximal effort).

### TMS task

The neurophysiological investigation was carried out in an additional task, called TMS-task, specifically devised to exclude all the possible bottom-up confounding factors that could influence corticospinal excitability, such as the actual force level, joint velocity and background electromyographic activity. This task was executed by all the subjects soon after the main motor task and consisted in a red line visible on the PC monitor, that represented the 30% of the subject’s MVF (Fig 1B). The subjects were asked to keep the cursor stable on the red line until its color changed from yellow to green (30 ± 1% MVF). Only when the cursor was stable for at least 500 ms, the software automatically triggered the TMS pulse, which was delivered on the FDI optimal scalp position at 100% of the resting motor threshold (rMT). For each subject, the intensity of stimulation remained stable across the sessions, thus ruling out any effect of TMS intensity on the changes of MEP amplitude and CSP duration. The TMS-task consisted of 16 trials, each one lasting 5000 ms. If the subject was not able to maintain the cursor stable on the red line, the TMS pulse was not delivered and the trial was repeated again. Hence, the task was repeated until 16 TMS pulses were obtained. In order to avoid adaptation, the TMS pulse could be triggered randomly between 500 ms and 1300 ms after the cursor had become green. In each trial, electromyographic (EMG) activity was recorded 100 ms before and 1000 ms after the TMS pulse. A 10-seconds interval was inserted between the trials. The TMS task was performed about 7–8 minutes after the application of TENS.

### TMS stimulation and EMG recording

Surface EMG was recorded from the motor point of the FDI and abductor digiti minimi (ADM) muscles of the right hand with bipolar self-adhesive Ag-AgCl electrodes (1.5×2.5 cm) in a belly-tendon montage. The ground electrode was attached to the wrist. EMG signals were band-pass filtered (20 Hz-2.5 kHz; plus 50-Hz notch) (D360, Digitimer, Welwyn Garden City, UK), amplified at a gain of 1000 (Digitimer, Hertfordshire, England), digitized at 5 kHz with laboratory interface (Cambridge Electronic Design 1401, Cambridge, England) controlled by Spike 2 (version 6, Cambridge Electronic Design) and then analyzed off-line.

A figure-of-eight coil (outer diameter of each wing 110 mm) was used to apply a biphasic single TMS pulse (STM 9000 magnetic stimulator, Ates-EBNeuro, Italy). The coil was mounted on an articulated arm and positioned tangentially to the skull at an angle of 45° to the sagittal plane [23, 24]. The FDI optimal scalp position was identified by moving the coil in small steps laterally to vertex in the left hemisphere and by delivering TMS pulses with constant intensity until stable and maximal MEPs could be evoked in the relaxed FDI muscle. The rMT was defined as the lowest stimulus intensity able to evoke MEPs with an amplitude of at least 50 μV in at least five out of ten trials in the FDI muscle.

Peak-to-peak MEP amplitude was recorded from the two muscles (FDI_amp_ and ADM_amp_) and the duration of the cortical silent period (CSP) was recorded from the active muscle (FDI_csp_). The CSP duration was calculated from the onset of the TMS trigger pulse and the moment in which the rectified EMG activity, averaged over a 10 ms period, had returned to 50% of pre-stimulus values [25]. Pre-stimulation EMG level was evaluated by calculating the root mean square of the background EMG over 50 ms prior to MEP onset. Trials in which the FDI MEP amplitude was lower than the mean background EMG were removed [26]. Moreover, all the neurophysiological data were inspected to rule out outliers (i.e., values 2×SD above or below the mean value for each subject in each session). Following these procedures, 6.27% of trials were removed in the experimental group and 7.5% in the control group.

MEP amplitude from the FDI and ADM were recorded also with subjects at rest (FDI_amp_rest_ and ADM_amp_rest_), by stimulating at 120% rMT at the beginning (10 trials) and at the end (10 trials) of whole experimental procedure. All the trials in which the root mean square of the background EMG of the FDI and ADM muscles was > 10 μV were removed [27]. Additionally, also the neurophysiological data recorded at rest were checked to exclude outliers. Following these procedures, 2.94% of trials were removed in the experimental group and 2.64% in the control group.

### Data analysis

Before calculating the mean value of Force_peak_ in each session for each subject, we removed the trials in which volunteers did not press the piston after having pressed the mouse key (2.13% for the experimental group and 1.35% for the control group).

Analyses were performed using SPSS Statistics 21 software (SPSS Inc). Repeated measures analyses of variance (rmANOVAs) were carried out to assess the effects of group (between-subject factor, 2 levels: experimental vs. control) and sessions (within-subject factor, 3 levels: baseline, manipulation, final) with regards to the behavioral parameters (normalized Force_peak_, Strong_press_, FI), subjective parameters (perception of force and sense of effort) and neurophysiological parameters (MEP, CSP and background EMG). Independent samples t-test was applied to compare across groups the level of expectation (NRS scores), the judgments about the effects of TENS (VAS scores). In all the analyses, post-hoc comparisons were executed by means of 2-tailed t-tests for paired or independent samples, using the Bonferroni correction for multiple comparisons where necessary. The level of significance was set at *p* < 0.05. All the data are expressed as mean ± SE.

## Results

MVF as measured in the initial calibration phase did not differ between the two groups (experimental: 21.74 ± 0.88 N, control: 20.78 ± 0.81 N, independent sample t-test, t_(30)_ = 0.80, p = 0.431).

### Behavioral data

**Fig 2:**
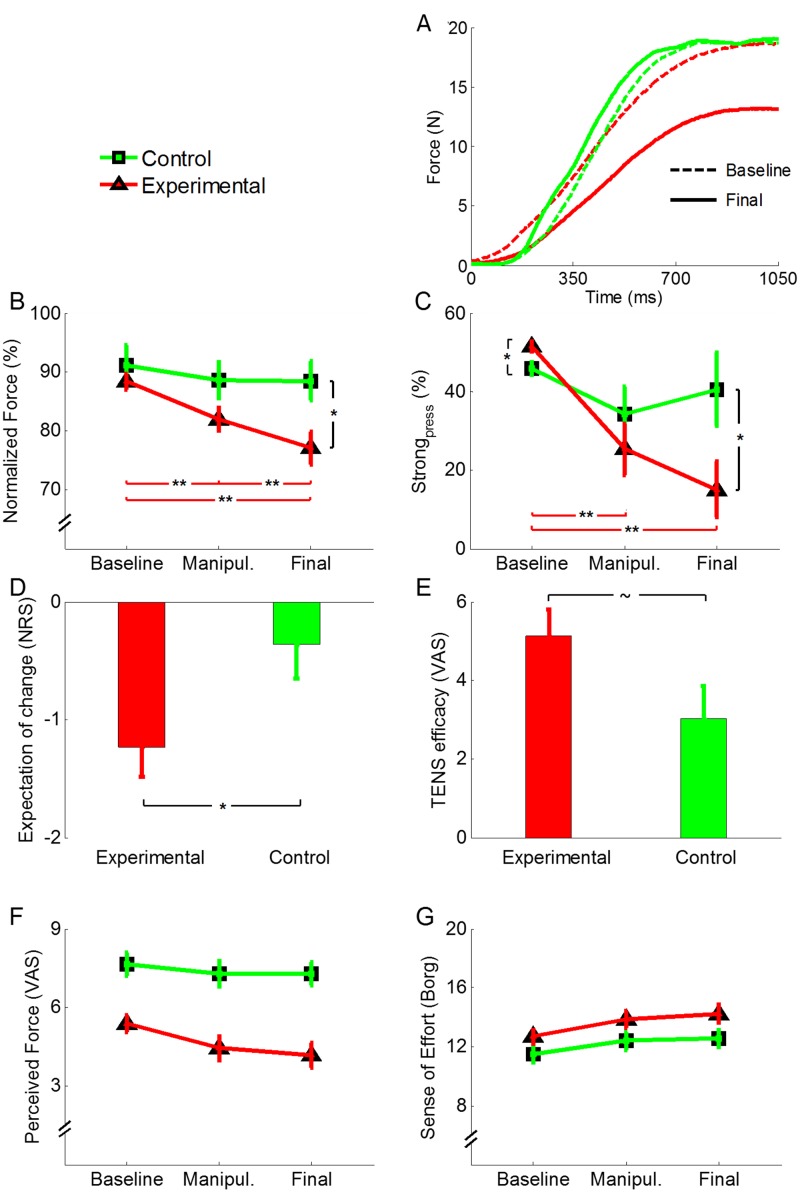
Behavioral and subjective data. A) Force profile (average of 50 trials) of the baseline (dashed lines) and final (solid lines) sessions in one subject of the experimental group (red lines) and one subject of the control group (green lines). B) Normalized force peak decreases in the experimental group (red line) from baseline to final session, whereas it remains stable in the control group (green line). Moreover, the two groups have nearly different values in the final session. C) Also the percentage of strong pressures decreases in the experimental group (red line) from baseline to final session, whereas it remains stable in the control group (green line). D) Scores of expectation of change in performance. The experimental group (red bar) expected a more negative change of performance than the control group (green bar). E) Judgments of treatment efficacy. The experimental group (red bar) has higher score than the control group (green bar). F) Subjective perception of force. In general, the experimental group (red line) felt weaker than the control group (green line). G) Sense of effort [22] was overall higher in the final than in the baseline session. All the values are expressed as mean ± SE. **p < 0.010, *p < 0.050, ~ p = 0.056.

ANOVA on normalized Force_peak_ revealed a significant effect of session (F_(2,60)_ = 15.16, p < 0.001), due to lower values in the final (82.77 ± 2.19) compared to the baseline (89.76 ± 1.74; p < 0.001) session. The factor group revealed a non-significant trend (F_(1,30)_ = 3.84, p = 0.059), due to lower values in the experimental group (82.45 ± 2.44) compared to the control group (89.42 ± 2.59). More interestingly, the interaction session × group was significant (F_(2,60)_ = 5.71, p = 0.005). Post-hoc comparisons showed that the experimental group was significantly weaker in the final (77.04 ± 2.82%) than in the manipulation (81.94 ± 2.02%) and baseline (88.36 ± 1.41%) session (p = 0.008 and p < 0.001, respectively). Moreover, the experimental group was also weaker in the manipulation than in the baseline session (p = 0.002). Conversely, in the control group no significant difference was found between the final (88.5 ± 3.39%), the manipulation (88.61 ± 3.13%) and the baseline (91.15 ± 3.36%) sessions (for all comparisons, p > 0.509). On the other hand, the two groups had different force values in the final (p = 0.014), but not in the baseline (p = 0.430) and manipulation (p = 0.077) sessions (Fig 2A and 2B).

ANOVA on Strong_press_ disclosed a similar pattern of results with a significant effect of session (F_(2,60)_ = 10.09, p < 0.001), due to lower values in the final (27.77 ± 5.78) than in the baseline session (48.73 ± 1.08; p = 0.002), but no effect of group (F_(1,30)_ = 2.25, p = 0.144). The interaction session × group was significant (F_(2,60)_ = 4.58, p = 0.014). Post-hoc comparisons showed that the experimental group pressed the piston more frequently weaker in the final (14.98 ± 7.16%) and in manipulation (25.29 ± 6.53) compared to the baseline (51.52 ± 1.47%) session (p < 0.001 and p = 0.004, respectively) (Fig 2C). No difference was found in the control group between the final (40.54 ± 9.25%), the manipulation (34.27 ± 7.05%) and the baseline (45.94 ± 1.6%) sessions (for all comparisons, p > 0.391). Moreover, in the final session the experimental group had significantly lower values than the control group (p = 0.035). Conversely, in the baseline session the experimental group had significantly higher values than the control group (p = 0.015), while there was no difference between groups in the manipulation session (p = 0.358). Altogether, these findings suggest that the proposed procedure was successful in inducing a decrease of force production in the experimental group.

Analysis of FI, revealed no significant effect of session (F_(2,60)_ = 0.376, p = 0.688), group (F_(1,30)_ = 0.016, p = 0.901) or session × group interaction (F_(2,60)_ = 0.102, p = 0.903), suggesting that the index of fatigue extracted from our data was similar in the two groups and across sessions (Fig 5B).

### Subjective data

Analysis of the expectation level showed a significant difference between the two groups (t_(30)_ = -2.31, p = 0.028), due to lower values in the experimental (-1.24 ± 0.25) compared to the control group (-0.37 ± 0.28) (Fig 2D). Scores on TENS efficacy were slightly higher in the experimental (5.14 ± 0.66) than in the control group (3.02 ± 0.85) (t_(30)_ = 1.99, p = 0.056) (Fig 2E).

Analysis of force perception disclosed a significant effect of session (F_(2,60)_ = 8.80, p < 0.001), due to overall lower values in the final (5.73 ± 0.33) compared to the baseline session (6.52 ± 0.26; p = 0.002) and a significant effect of group (F_(1,30)_ = 22.44, p < 0.001), due to lower values in the experimental (4.67 ± 0.40) compared to the control group (7.40 ± 0.42). The session × group interaction was not significant (F_(2,60)_ = 2.29, p = 0.110).

Analysis of the sense of effort revealed a significant effect of session (F_(2,60)_ = 11.12, p < 0.001), due to higher values in the final (13.47 ± 0.43) compared to the baseline (12.07 ± 0.38; p = 0.001) session. Group (F_(1,29)_ = 3.71, p = 0.064) and the interaction session × group (F_(2,60)_ = 0.54, p = 0.586) were not significant (Fig 2G).

### Neurophysiological data

Independent sample t-test on the rMT of the two groups (experimental group: 59.65 ± 10.79, control group: 64.13 ± 8.98) revealed no significant differences (t_(30)_ = -1.27, p = 0.214).

**Fig 3:**
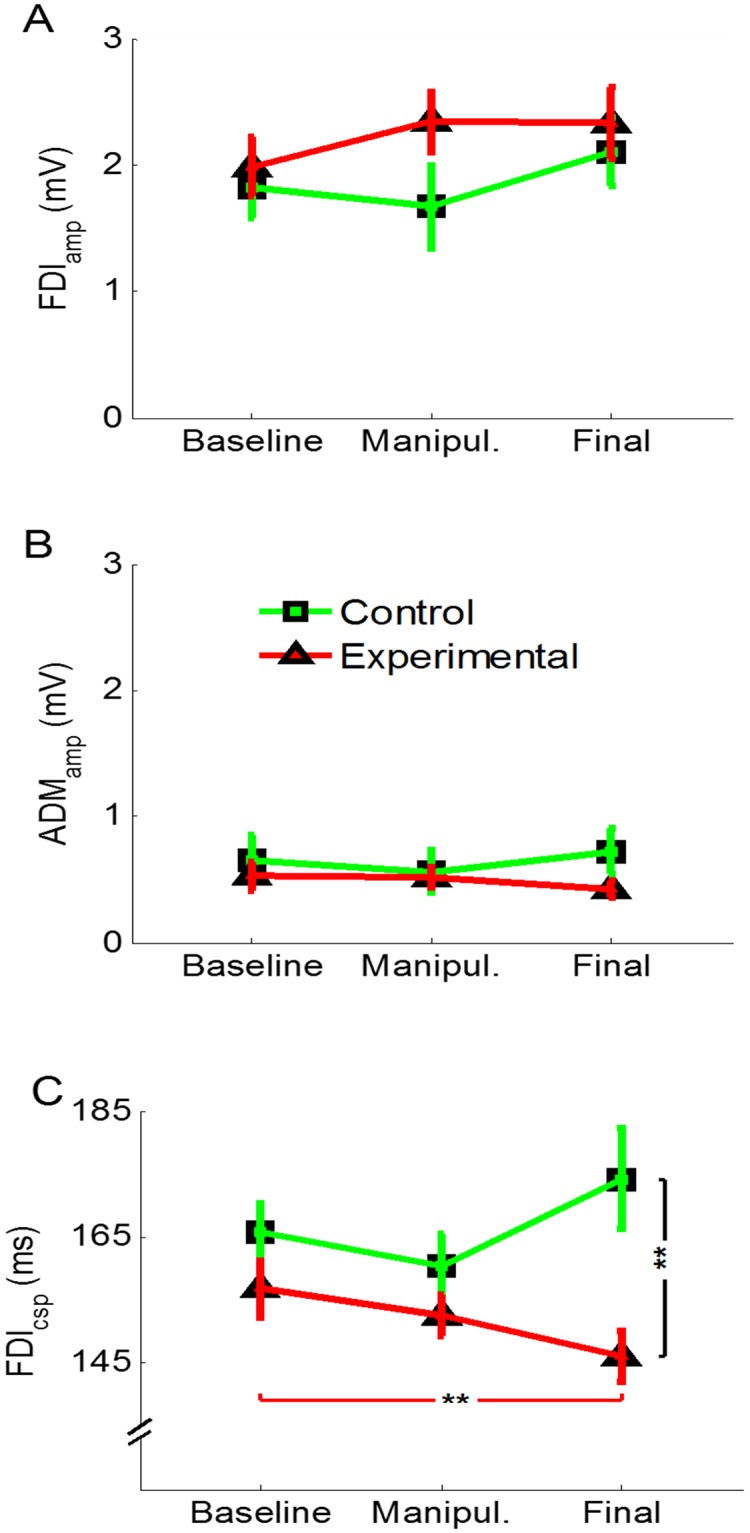
Neurophysiological data. A) MEP amplitude recorded from the FDI muscle was comparable in the experimental (red line) and the control (green line) and across sessions. B) MEP amplitude recorded from the ADM muscle was comparable in the experimental (red line) and the control (green line) and across sessions. C) Duration of the CSP was shorter in the final than in the baseline session in the experimental group (red line), whereas there was a slight increase, although not significant, in the control group (green line). Moreover, the two groups had different CSP duration in the final session. All the values are expressed as mean ± SE. **p < 0.010.

**Fig 4:**
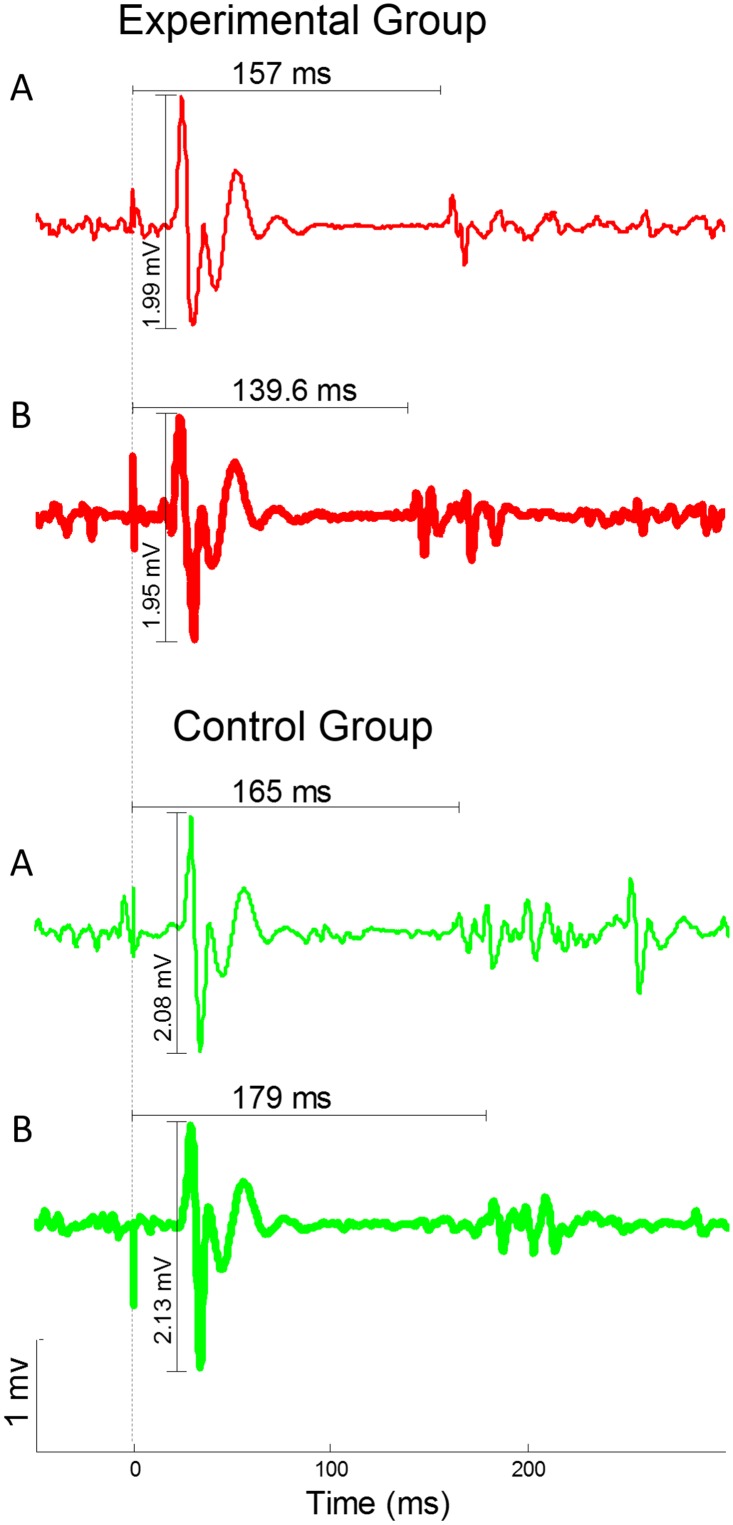
Representative neurophysiological traces recorded from the FDI muscle. The traces represent the average of the 16 trials of the TMS task in the baseline session (A, thin lines) and in the final session (B, thick lines). In red are represented the traces of one subjects of the experimental group. In green are represented the traces of one subject of the control group. The TMS pulse was delivered at 0 ms (dashed line).

ANOVA on the FDI_amp_ revealed no effect of session (F_(2,60)_ = 1.70, p = 0.191), no effect of group (F_(1,30)_ = 1.19, p = 0.284), and no significant session × group interaction (F_(2,60)_ = 1.31, p = 0.278) (Fig 3A). ANOVA on the ADM_amp_ revealed no effect of session (F_(2,60)_ = 0.38, p = 0.683), no effect of group (F_(1,30)_ = 0.66, p = 0.422), and no session × group interaction (F_(2,60)_ = 2.24, p = 0.116) (Fig 3B). Analysis of the FDI_csp_ revealed no effect of session (F_(2,60)_ = 1.09, p = 0.342), but a significant effect of group (F_(1,30)_ = 6.67, p = 0.015), due to lower values in the experimental (151.78 ± 4.0) than in the control group (166.76 ± 4.22). The session × group interaction was also significant (F_(2,60)_ = 5.39, p = 0.007). Post-hoc comparisons showed that the experimental group presented shorter FDI_csp_ duration in the final (146.17 ± 3.94 ms) than in the baseline (156.76 ± 4.39 ms; p = 0.005) session, but no difference was found between the manipulation (152.41 ± 3.29) and the other two sessions (for both comparisons, p > 0.100) (Fig 3C). Moreover, no difference was found in the control group between the final (174.27 ± 7.94), the manipulation (160.33 ± 5.02) and the baseline (165.67 ± 4.7) sessions (for all comparisons, p > 0.203). In the final session, the FDI_csp_ duration of the experimental group was different from the control group (p = 0.003). Representative EMG traces of one subject for each group are illustrated in Fig 4 for the baseline and final sessions.

**Fig 5:**
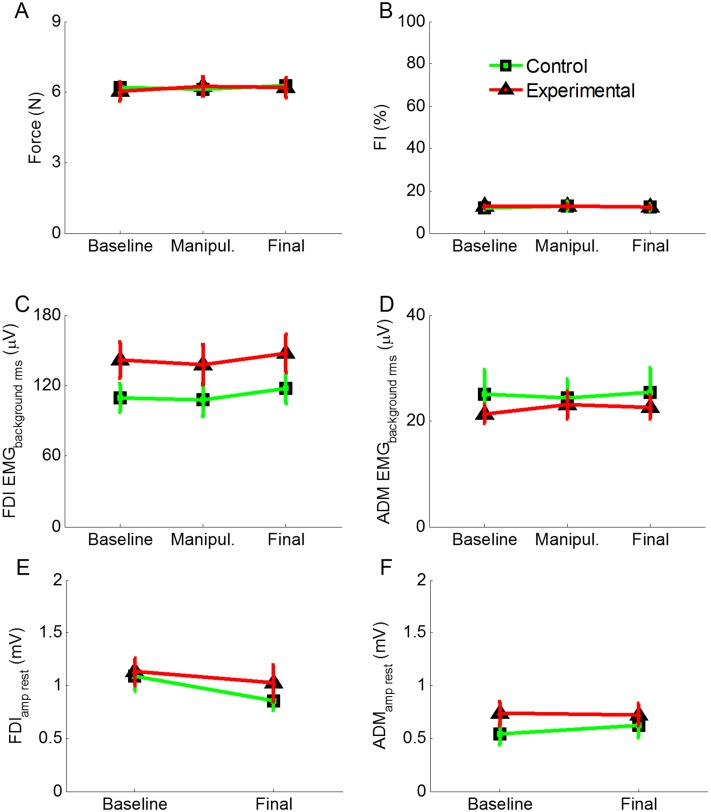
Additional parameters recorded during the TMS-task and at rest. A) Force level of the two groups of participants in the TMS-task, corresponding to the moment in which the TMS pulse was delivered. B) Index of fatigue (FI). The experimental (red line) and control group (green line) had similar FI values across the sessions, ruling out the effect of fatigue. C) EMG background activity recorded from the FDI muscle before the TMS pulse. D) EMG background activity recorded from the ADM muscle before the TMS pulse. E) MEP amplitude recorded at rest from the FDI muscle. F) MEP amplitude recorded at rest from the ADM muscle. All the data are represented as mean ± SE.

In order to rule out whether the MEP amplitude was a mere reflection of the amount of applied force, we analyzed (rmANOVA) the level of the force of each group in the three sessions (baseline, manipulation, final) of the TMS-task, that is in the moment in which the TMS pulse was applied. This analysis showed no effect of session (F_(2,60)_ = 0.65, p = 0.526), no effect of group (F_(1,30)_ = 0.01, p = 0.913) and no session × group interaction (F_(2,60)_ = 0.97, p = 0.384), suggesting that during the TMS-task, the force level was the same in both groups and all the sessions (Fig 5A). This suggests that the MEP amplitude was not influenced by the level of force exerted at the time of the TMS pulse. In order to control whether the MEP amplitude was influenced by the different activation in the preceding EMG activity, we analyzed the EMG background activity before the MEP onset. This analysis disclosed, no effect of session (FDI: F_(2,60)_ = 1.05, p = 0.357; ADM: F_(2,60)_ = 0.13, p = 0.874), no effect of group (FDI: F_(1,30)_ = 2.47, p = 0.127; ADM: F_(1,30)_ = 0.33, p = 0.568) and no session × group interaction (FDI: F_(2,60)_ = 0.017, p = 0.983; ADM: F_(2,60)_ = 0.31, p = 0.738), suggesting that EMG background activity was similar in both groups and all the sessions and therefore the MEP amplitude was not influenced by differences in preceding EMG activity (Fig 5C and 5D).

Finally, we analyzed the neurophysiological data recorded at rest, that is before starting the whole protocol and after its completion. ANOVA on FDI_amp_rest_ and on ADM_amp_rest_ disclosed no effect of session (FDI_(1,30)_ = 3.15, p = 0.086; ADM: F_(1,30)_ = 0.564, p = 0.459), no effect of group (FDI_(1,30)_ = 0.3, p = 0.595; ADM: F_(1,30)_ = 0.631, p = 0.433) and no session × group interaction (FDI: F_(1,30)_ = 0.21, p = 0.654; ADM: F_(1,30)_ = 0.882, p = 0.355) (Fig 5E and 5F). In order to control the preceding EMG activity at rest, we analyzed the EMG background activity before the MEP onset of the two groups and in the two sessions. This analysis disclosed no effect of session (FDI: F_(1,30)_ = 2, p = 0.167; ADM: F_(1,30)_ = 0.01, p = 0.942), no effect of group (FDI: F_(1,30)_ = 1.85, p = 0.184; ADM: F_(1,30)_ = 0.006, p = 0.941) and no session × group interaction (FDI: F_(1,30)_ = 0.83, p = 0.369; ADM: F_(1,30)_ = 0.010, p = 0.921).

## Discussion

This study tackled the behavioural and neurophysiological correlates of the nocebo effect in motor performance. The behavioural data proved that the nocebo procedure was successful in inducing a reduction of force. With regard to the neurophysiological data, two main findings emerged: First, following the nocebo procedure only the CSP duration was modulated, whereas MEP amplitude remained stable; second, the duration of the CSP was reduced. We will discuss these findings, by taking into account our previous study on the placebo effect [14].

### Force decreases following a nocebo procedure

The behavioural results showed a pronounced reduction of force in the experimental group compared to the control group. Both the normalized force peak (that refers to the general performance in relation to the MVF) and the percentage of strong pressures (that refers to the number of times in which a subject consistently presses the piston above a certain threshold value) decreased across sessions only in the experimental group.

Since motor performance in the control group did not decline, the finding in the experimental group cannot be explained by massed repetition of the task [28] or by a physiological reduction of force [29, 30]. Instead, the marked reduction of force was likely induced by the nocebo procedure. Subjects of the experimental group were told that TENS would have decreased their force and the conditioning procedure was applied in order to make them believe that TENS was really effective. By surreptitiously reducing the cursor’s excursion range, subjects could see the cursor reaching lower lines of the target zone than before and were therefore conditioned about the effects of TENS in reducing force. Interestingly, in the final session—when the surreptitious reduction of the cursor was removed—subjects of the experimental group did not show an increase of force compared to the manipulation session, suggesting that force continuously decreased in this group.

The lower scores at the expectation scale given by the experimental group compared to the control group, together with higher scores of TENS efficacy, confirm that the nocebo procedure induced a subjective state characterized by the expectation of a worse outcome.

By comparing these findings to our previous study on the placebo effect, we can observe an opposite pattern of behaviour: increase of force in the placebo study [14] and reduction in the current study. This confirms that opposite verbal instructions and conditioning procedures result in divergent behavioural outcomes.

### The neurophysiological correlates of the nocebo effect in motor performance

In our previous paper on the placebo effect [14], the most evident neurophysiological findings were obtained in the group of participants who underwent a conditioning procedure in addition to verbal suggestion. For this reason, in the present study on the nocebo effect, we included also a conditioning phase. The prediction to find an opposite pattern of corticospinal activation between nocebo (current study) and placebo effect [14] was not confirmed, as MEP amplitude was not reduced and CSP duration was not increased by the nocebo procedure.

Among cognitive factors that can induce a reduction of MEP amplitude there is attentional decrease [31]. Hence, we suggest that MEP amplitude in our study did not decrease—despite the reduced motor performance in the experimental group—probably because the procedure prevented a reduction of attention, thus maintaining MEP size stable in both groups throughout the sessions. This raises the question why the placebo procedure resulted in a change of MEP amplitude [13], whereas here the nocebo procedure leaved MEPs unchanged. One possible and speculative answer is that being the placebo-induced expectation directed to a potential benefit, it is also associated to reward mechanisms [32–36] that can increase MEP amplitude [37]. Conversely, the nocebo procedure does not induce anticipation of reward and this fact, together with a constant level of attention throughout the sessions, could have maintained MEP amplitude stable.

Despite stable MEPs, the CSP duration changed, suggesting that only the CSP is modulated by the nocebo procedure. Changes in CSP duration have been previously described in healthy participants during the execution of specific motor tasks, like a visuomotor task [38], skilled hand actions [39] and strength training [40, 41]. In our study, however, the motor task was the same in the baseline and final sessions and in both groups, and therefore the CSP changes cannot be ascribed to the nature of the motor task itself. We can also exclude that the CSP reduction found in the current study was related to fatigue, because (i) to the best of our knowledge, exhaustive muscle activity induces CSP prolongation [42]; (ii) the index of fatigue extracted from our data was not different in the two groups and across the sessions; (iii) the influence of bottom-up factors—like the amount of force or movement speed—at the time of the TMS pulse was excluded, thus ruling out the impact of different sensory feedback from the contracting muscle on the CSP duration [41].

One possible explanation for the shorter CSP in the experimental group could be related to the difference in force perception between the manipulation and the final session. More precisely, it could be argued that during the conditioning phase, in which there was a surreptitious reduction of the cursor’s excursion range, subjects experienced lower force levels and higher sense of effort compared to the final session, in which instead the cursor’s excursion range was not manipulated. Consequently, the perception of the task to be easier in the final session could have been rewarding and this could explain a reduction in CSP. This explanation, however, does not appear to be supported by our data. First, the CSP duration was not significantly different between the final and manipulation session. Second, if the subjects had experienced the task to be easier in the final than in the manipulation session, we should have observed also a reduction of the sense of effort and an increase in force perception and force production. This was not the case, since subjective perception of force and sense of effort did not change from the manipulation to the final session, whereas the force level decreased.

The CSP duration was clearly shorter in the experimental group, whereas in the control group there was an opposite tendency toward a CSP prolongation, although not significant. Since TENS was applied exactly in the same way in the two groups, the different changes in CSP cannot be explained by the effects of TENS *per se*. Instead, the main factor distinguishing the two groups was the nocebo procedure, consisting of verbal suggestion and conditioning. The core of this procedure is an induced expectation of change in performance, and therefore we propose that this cognitive function may be responsible for the CSP reduction. Only few studies documented a change of CSP duration associated to cognitive functions. A study on action observation found specific modulation of the CSP when healthy participants observed actions attributed either to the self or to others [43], and another study described CSP changes due to attention toward the hand [44]. Interestingly, also the type of instructions given to the participants prior to the execution of a motor task modulates the CSP duration [45]. More precisely, the CSP was prolonged when subjects were instructed to relax the muscle after the TMS pulse, whereas it was shorter when they were instructed to contract [45]. Since the TMS pulse was delivered before the actual execution of the instructions, the observed changes in CSP represent the different instruction sets [45]. Although we used a different paradigm and our participants were required to maintain a constant contraction before and after the TMS pulse, we cannot exclude that the different instructions about TENS given to the two groups could have biased not only their expectation of change in performance but also their preparation to the TMS pulse. Hence, higher expectation of change could have induced alertness to the TMS pulse in the experimental group, thus shortening the CSP, whereas lower expectation of change could have induced a more relaxed attitude to the TMS pulse in the control group, thus slightly prolonging the CSP.

Even though this interpretation remains to be proven, our study clearly adds further evidence for a cognitive modulation of the CSP, by showing a reduction following a nocebo procedure. This result is similar to the one we previously observed for the placebo effect [14], suggesting that nocebo and placebo effects in motor performance share some common mechanisms. This means that, independently of the direction of the behavioural outcome (increase or decrease of force) or of the belief (positive or negative), the nocebo/placebo-induced expectation modulates the CSP in the same way. It could be speculated that probably the CSP is more easily modulated than the MEP. Support to this idea derives from a study demonstrating that the CSP threshold is lower than the MEP threshold, likely because of a more widespread placement of inhibitory neurons in the primary motor cortex that generate the CSP than of cortical interneurones that contribute to the generation of the MEP [46]. Of note, while MEP is related to excitatory circuits and can be influenced by the activity of both cortical and spinal neurons [47], the CSP is related to the activity of inhibitory motor circuits and, at least in the last part, is mainly generated by cortical mechanisms [18, 48]. Hence, the CSP reduction in the experimental group hints at a change in the cortical inhibitory circuits. The functional significance of the involvement of inhibitory circuits in the nocebo/placebo effects in motor performance, as well as the underlying mechanisms, remain to be explained. Potentially, a brain network involved in expectation and anticipation [5, 9, 49] may exert a top-down modulation on the inhibitory circuits of the primary motor area. As a consequence, the reduced inhibitory activity could represent a state of alertness of the motor system, that serves to deal with the expected changes of performance.

To summarize, beside a clear behavioural nocebo effect, the picture of the neurophysiological data was less clear-cut: While MEP amplitude remained stable, CSP duration was reduced after the nocebo procedure. The CSP reduction resembled the finding of our previous study in which a similar paradigm was applied to induce a placebo effect [14]. This suggests that expectation of a change in performance may engage cortical inhibitory activation in the nocebo and placebo effects, independently of the direction of the behavioural outcome. Although the technique of single-pulse TMS adopted in our study is suitable to measure changes in CSP duration [39, 40], it does not allow to go deeper into more fine-tuned changes of cortical inhibition. Likely, neurophysiological differences between placebo and nocebo effects in motor performance could be uncovered with more specific methods, like paired-pulse TMS or theta burst stimulation, that allow to stimulate selective cortical circuits within the motor cortex.
